# Supplementary material for: Epithelial-to-mesenchymal transition and live cell extrusion contribute to measles virus release from human airway epithelia
Source: J Virol. 2025 Jan 10;99(2):e01220-24. doi: 10.1128/jvi.01220-24 (PMC11852777; doi:10.1128/jvi.01220-24)

Fig S1. Characterization of ISG-high cells.

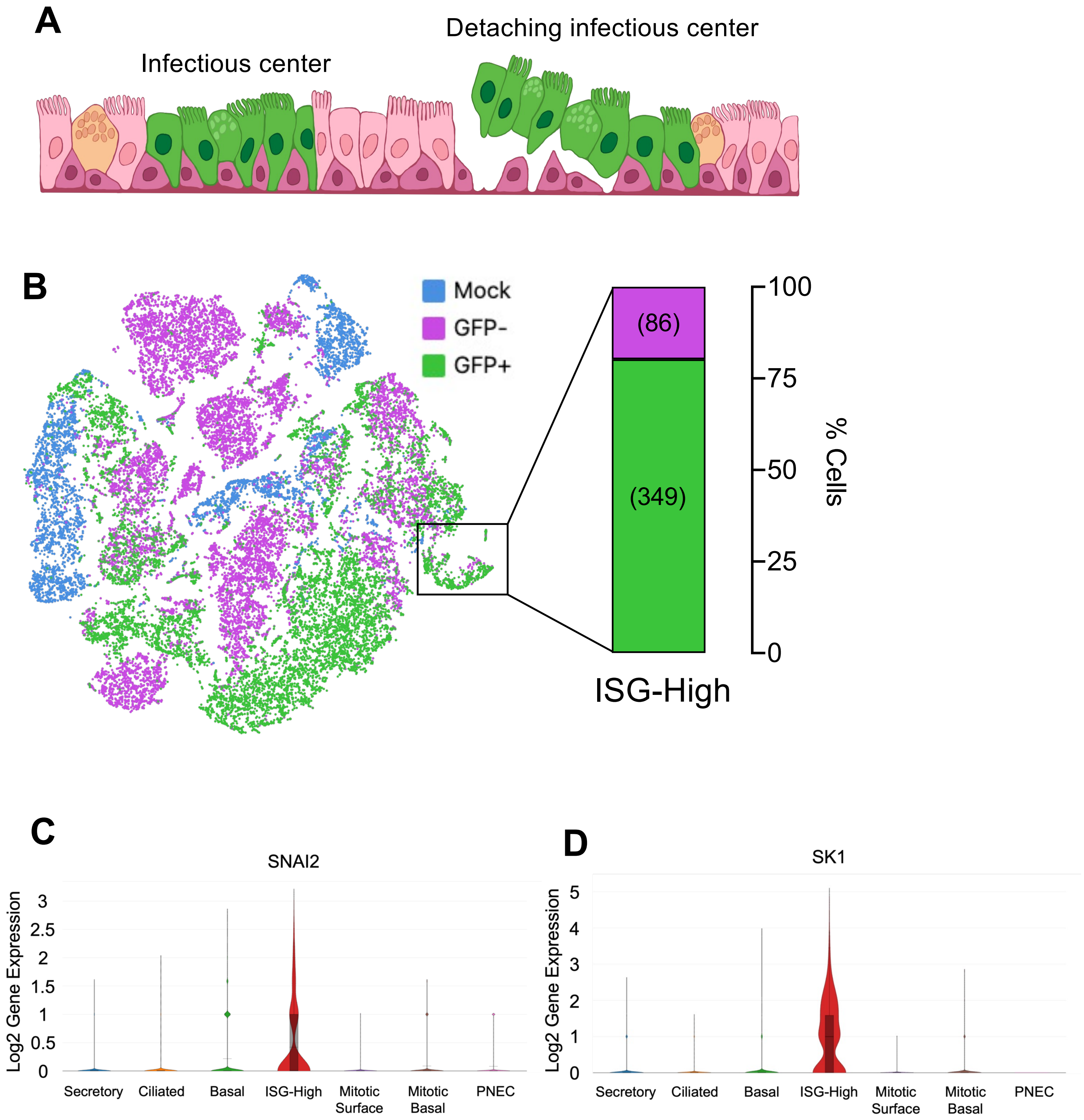

Fig S2. qRT-PCR confirmation of scRNA-seq.

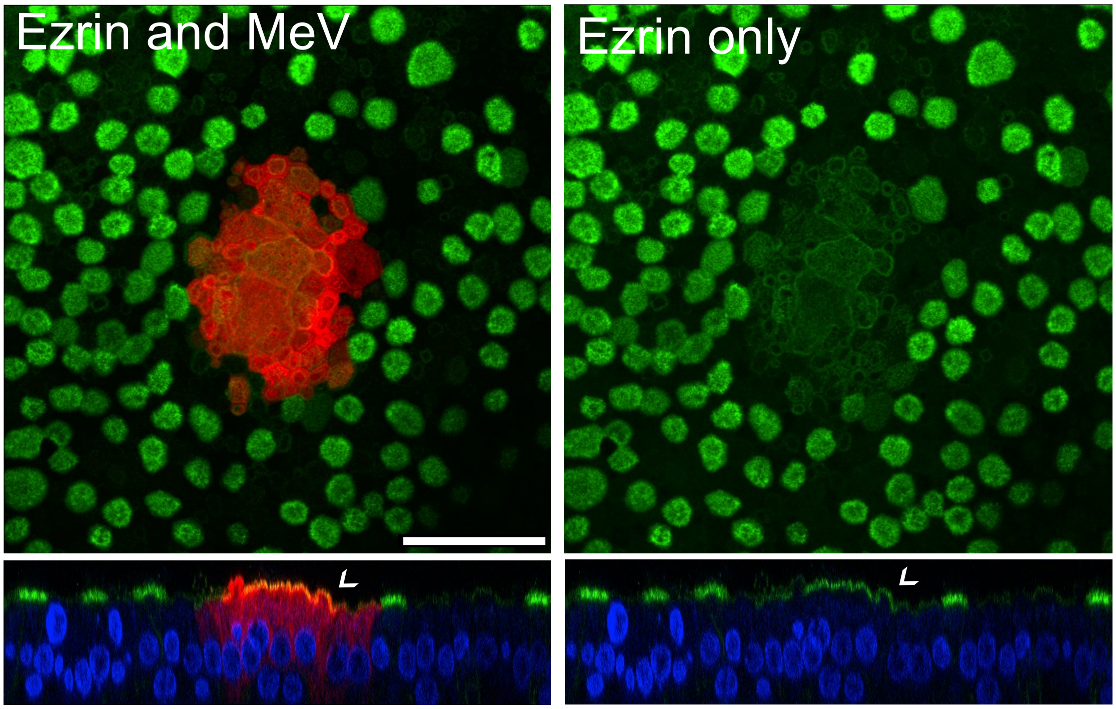

Fig S3. Cell type distribution of differentially expressed LCE genes.

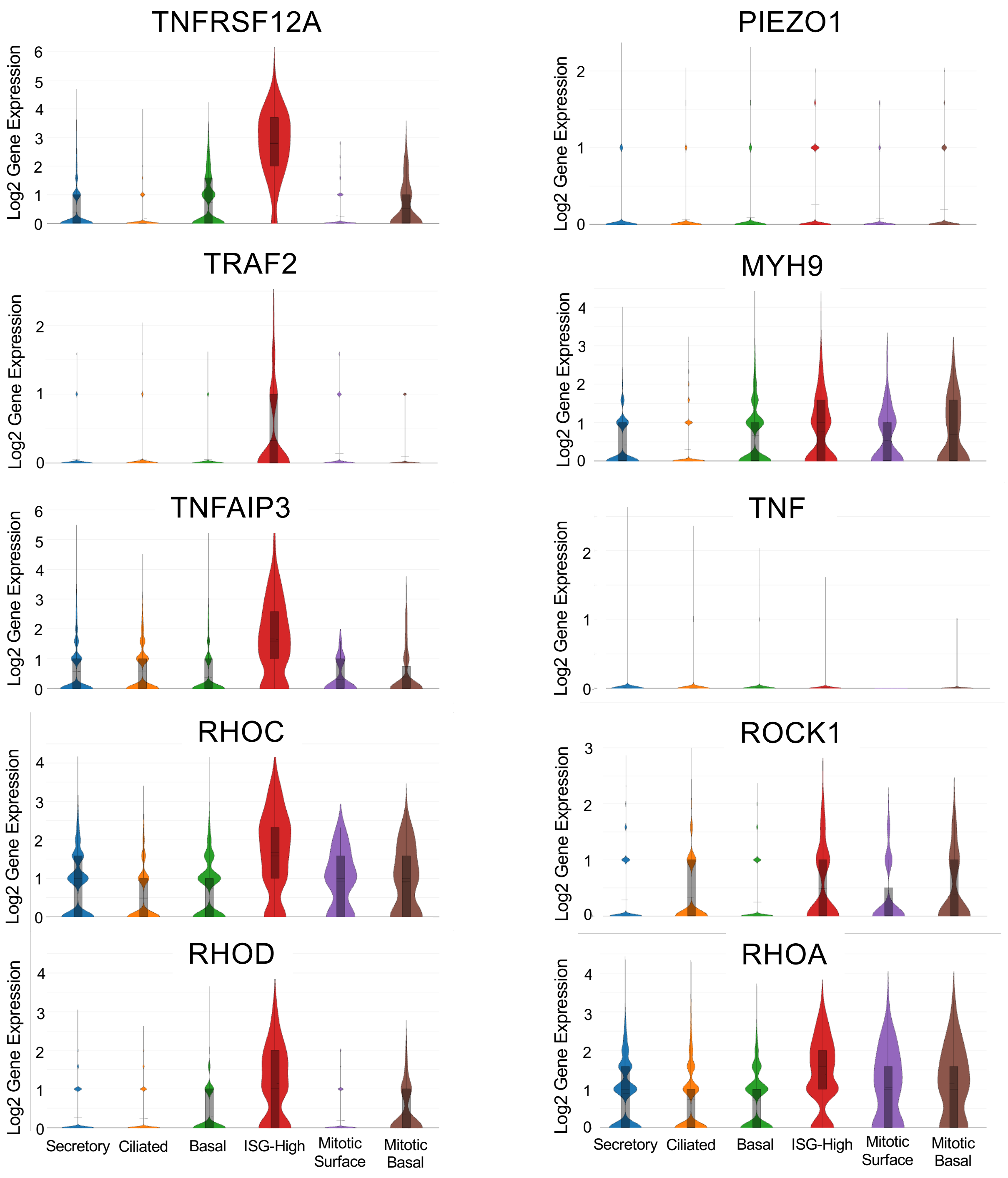

Supplement: Supplemental figures — Figures S1 to S3. [file jvi.01220-24-s0001.pdf]
