# Supplementary material for: Epithelial-to-mesenchymal transition and live cell extrusion contribute to measles virus release from human airway epithelia
Source: J Virol. 2025 Jan 10;99(2):e01220-24. doi: 10.1128/jvi.01220-24 (PMC11852777; doi:10.1128/jvi.01220-24)
Supplement: Supplemental legends — Legends for Fig. S1 to S3. [file jvi.01220-24-s0002.pdf]

Fig S1. Characterization of ISG-high cells. (A) Schematic depiction of infectious center formation and detachment. Green cells represent MeV-infected cells. Cell-to-cell spread of MeV over 3-5 days results in an infectious center, left. Beginning around 5 days post-infection, infectious centers begin to detach as a unit from the rest of the epithelial layer, right.
(B) The t-SNE plot from Fig 1 with GFP status overlayed. GFP+ indicates infected cells, GFP- indicates uninfected cells from MeV-infected HAE. Mock indicates cells mock infected. The box highlights the ISG-high cell cluster. The adjoining graph identifies the division of ISG-high cells that are GFP+ or GFP-. (C) Violin plot of SNAI2 expression across cell clusters. (D) Violin plot of SK1 expression across cell clusters.

Fig S2. Ezrin expression. HAE infected with MeV-mCherry were fixed at 7 days post-infection and immunostained for ezrin. A z-stack image is shown below. The arrowhead points to ezrin staining that has been disturbed at the surface of the infectious center. Blue = DAPI. Scale bar = 50 μm.

Fig S3. Cell type distribution of differentially expressed LCE genes. The violin plots of the Figure 5B heat map genes are shown.
