# Supplementary material for: Epithelial-to-mesenchymal transition and live cell extrusion contribute to measles virus release from human airway epithelia
Source: J Virol. 2025 Jan 10;99(2):e01220-24. doi: 10.1128/jvi.01220-24 (PMC11852777; doi:10.1128/jvi.01220-24)
Supplement: Table S1 — Primer sequences for qRT-PCR analysis. [file jvi.01220-24-s0003.docx]

**S1 Table. Primer sequences for qRT-PCR analysis.**

| Gene name | Direction | Sequence |
| --- | --- | --- |
| *MMP1* | Forward | CCTAGTCTATTCATAGCTAATCAAGAGGATGT |
|  | Reverse | AGTGGAGGAAAGCTGTGCATAC |
| *MMP10* | Forward | ATCTGAGATGCCAGCCAAGT |
|  | Reverse | AGGGTTCCAGTGGGATCTTC |
| *TGFB1* | Forward | GGCCTCCCCACCACACCAG |
|  | Reverse | GCCGCAGCTTGGACAGGATC |
| *SNAI2* | Forward | ATCTGCGGCAAGGCGTTTTCCA |
|  | Reverse | GAGCCCTCAGATTTGACCTGTC |
| *SMAD3* | Forward | GCCTGTGCTGGAACATCATC |
|  | Reverse | TTGCCCTCATGTGTGCTCTT |
| *SFRS9* | Forward | TGCGTAAACTGGATGACACC |
|  | Reverse | CCTGCTTTGGTATGGAGAGTC |
